# Depressed sympathovagal modulation indicates sepsis in patients with suspected infection

**DOI:** 10.1097/MD.0000000000018961

**Published:** 2020-01-24

**Authors:** Ching-Tang Hsu, Henry Chih-Hung Tai, Jui-Yuan Chung, Jiann-Hwa Chen, Wei-Lung Chen

**Affiliations:** a Department of Emergency Medicine, Cathay General Hospital; b School of Medicine, Fu-Jen Catholic University, Taiwan.

**Keywords:** autonomic nervous function, heart rate variability, sepsis, sympathetic activity, vagal activity

## Abstract

This study explored whether sympathovagal modulation assessed through frequency domains of heart rate variability (HRV) can indicate sepsis in patients with suspected infection.

In total, 370 consecutive adult patients with suspected infection admitted to the emergency department were enrolled in this single-center cohort study. A continuous 10-minute electrocardiography for HRV analysis was recorded immediately for these patients after inclusion. Patients were stratified into non-sepsis and sepsis groups based on a sepsis-related organ failure assessment score of ≥2 that met the Third International Consensus Definitions for Sepsis. Seven frequency domains of HRV were compared between these 2 groups.

Compared with the non-sepsis group (n = 98), the sepsis group (n = 272) had a significantly lower incidence of respiratory tract infection, higher total power, higher very-low-frequency component, higher high-frequency (HF) component, higher normalized HF component, lower normalized low-frequency (LF) component, and lower LF component/HF component ratio (LF/HF). Multiple logistic regression model identified HF component (odds ratio [OR] = 0.994; 95% confidence interval [CI], 0.990–0.999) and LF/HF (OR = 0.494; 95% CI, 0.423–0.578) as significant variables associated with sepsis. The area under receiver operating characteristic curves of HF component and LF/HF was 0.741 (95% CI, 0.685–0.797) and 0.930 (95% CI, 0.900–0.960), respectively, in identifying sepsis in patients with suspected infection.

Tilted sympathovagal balance toward increased vagal activity and depressed sympathetic modulation, assessed by the HF component and LF/HF, may indicate sepsis in patients with suspected infection.

## 1 Introduction

The overall mortality and emergency department (ED) admission rates for sepsis have been estimated to be 30% to 50% and 14%, respectively,^[1–4]^ which demonstrate the seriousness of the disease. Because sepsis is one of the commonest reasons for hospital admission, patients with sepsis usually present themselves initially in the ED, and the speed of diagnosis and the appropriateness of therapy administered affect the outcome.^[1,5]^ Thus, the ability to accurately identify sepsis at ED is important. In February 2016, new criteria for sepsis, called the Third International Consensus Definitions for Sepsis and Septic Shock (Sepsis-3),^[6]^ were published in order to replace the previous criteria (Sepsis-1 and Sepsis-2).^[7,8]^ Sepsis is defined as a life-threatening organ dysfunction caused by a dysregulated host response to infection. Organ dysfunction is identified as an acute change in the total sepsis-related organ failure assessment (SOFA) score of ≥2 points consequent to the infection.^[6,9,10]^ The task force suggests that quick SOFA criteria be used to prompt clinicians to rapidly assess patients with suspected infection^[6,10]^ and further to confirm sepsis by evaluating whether the SOFA score of the patient is ≥2. One of the major concerns with the SOFA score is that it is complex and not a practical and available bedside tool, especially outside the intensive care unit (ICU).^[11–14]^ Furthermore, an Hour-One Bundle of Surviving Sepsis Campaign was proposed with the explicit intention of beginning resuscitation and management immediately.^[15]^ Consequently, considerable effort was devoted to improving our ability to rapidly assess patients with suspected infection by using a variety of predicting scores and clinical decision rules. Despite these efforts, none has emerged as a compelling definitive tool to identify sepsis in a short period.

Heart rate variability (HRV) analysis is a noninvasive tool that can evaluate autonomic nervous modulation of the heart. A power spectral analysis of HRV provides an assessment of the degree of sympathetic and parasympathetic modulation of the heart over a relatively short period.^[16–18]^ The power spectrum of HRV is often categorized into a high-frequency (HF) component and a low-frequency (LF) component. The HF component is related to respiratory sinus arrhythmia and cardiac vagal activity, whereas the LF component and normalized LF (LF%) component are jointly modulated through the neural activities of both vagal and sympathetic nerves. A power spectral analysis of HRV has gained popularity and has been broadly applied as a functional indicator of the autonomic nervous system.^[16–18]^ Moreover, a reduction in HRV and impaired sympathovagal balance, as represented by a depressed LF/HF component ratio (LF/HF), have been indicative of illness severity, development of multiple organ dysfunction syndrome, success or failure of early resuscitation, development of septic shock, and in-hospital mortality in patients with sepsis.^[19–22]^

Given the strong association between autonomic nervous modulation assessed using HRV and the disease severity of sepsis, this study explored whether the power spectrum of HRV can rapidly identify sepsis in patients with suspected infection.

## 2 Materials and methods

### 2.1 Study design

This was a prospective cohort study that investigated whether autonomic nervous modulation, as indicated by a power spectral analysis of HRV, can identify sepsis in adult patients with suspected infection in an ED. The study protocol was approved by the Institutional Review Board of Cathay General Hospital. Written informed consent was obtained from patients or their next of kin before enrolling them in the study. This study was performed in accordance with relevant guidelines and regulations.

### 2.2 Study setting and selection of participants

This study was conducted in an ED of a 700-bed university-affiliated medical center, with a 40-bed ED, staffed with board-certified emergency physicians that provide care for approximately 55,000 patients per year. From January 2018 to December 2018, adult patients (aged >18 years) with suspected infection were consecutively enrolled. Patients with coronary artery disease complicated with acute myocardial infarction (AMI) within 12 months, congestive heart failure (CHF) of functional class III to IV, and neuropathy or diagnosed autonomic dysfunction were excluded from this study because their autonomic nervous activity would be significantly affected by the underlying disease.^[16]^ Moreover, patients with persistent arrhythmia and cardiac pacing were excluded because HRV could not be analyzed in these patients.^[16]^ Additionally, patients with mechanical ventilation during electrocardiographic (ECG) recording were excluded because HRV would be affected by respiration fluctuation, especially HF.^[16]^

### 2.3 Study protocol and outcome measures

Under standard ED management, a 10-minute ECG recording (ECG 100C, ECG Amplifier, BIOPAC Systems, Inc, Goleta, Calif) was performed on patients in the supine position immediately after their enrollment, and output ECG signals were digitized using an A/D converter (MP150WSW, Starter System for Desktop and Notebook PCs, BIOPAC Systems). Digitized ECG signals were subsequently stored in a notebook computer for later HRV analysis. All procedures were performed in an air-conditioned resuscitation room with a constant temperature of approximately 25°C and suitable humidity.

The primary outcome of this study was sepsis that met the criteria of Sepsis-3.^[6,10]^ Sepsis is defined as life-threatening organ dysfunction caused by a dysregulated host response to infection. Organ dysfunction is identified as an acute change in total SOFA score of ≥2 points. The baseline SOFA score is assumed to be zero unless the patient has a preexisting (acute or chronic) organ dysfunction before the infection onset. The score grades abnormality on the basis of the organ system and accounts for clinical interventions, which is composed of 6 variables: respiration status (PaO_2_/FiO_2_ value), coagulation function (platelets count), liver function (bilirubin level), cardiovascular status (mean arterial pressure with/without vasopressor), central nervous system (Glasgow Coma Scale score), and renal function (creatinine level and urine output).^[6,10]^ Each variable is graded from 0 to 4 points and adds up to 24 points for the 6 variables. Clinical and laboratory data were checked and recorded for patients to complete the SOFA score. Patients were closely monitored and reevaluated for possible sepsis if clinically indicated when the initial SOFA score was <2. According to the outcome (i.e., sepsis), patients were categorized into 2 groups: non-sepsis and sepsis. Additionally, patients’ demographic information, underlying disease, and infection source were recorded using a formulated questionnaire. After hospital discharge, the in-patient medical record was reviewed to complete data collection. Study investigators who performed the HRV analysis were blinded to clinical information obtained and patient outcomes, and they did not influence clinical decision making.

### 2.4 HRV analysis

The method used to perform a power spectral analysis of HRV was described elsewhere^[22]^; it adhered to standards developed by the Task Force of the European Society of Cardiology and the North American Society of Pacing and Electrophysiology.^[16]^ In brief, digitized ECG signals were retrieved to measure consecutive RR intervals, which are the time intervals between successive pairs of QRS complexes, by using the software that was developed for the detection of the R wave (Matlab 6.5, MathWorks Inc., Natick, MA). All artifacts or ectopic beats were removed, and the resultant missing data (<5% per record) were replaced by interpolated beats derived from the nearest valid data. If the percentage of deletion was >5%, then the patient was excluded from the study. The last 512 stationary RR intervals were then used for the HRV analysis.

The power spectrum of these RR intervals was obtained through fast Fourier transformation (Mathcad 11, Mathsoft Inc., Cambridge, MA). The area under the spectral peaks within the range of 0.01 to 0.04 Hz, 0.04 to 0.15 Hz, 0.15 to 0.4 Hz, and 0.01 to 0.4 Hz was defined as the very-low-frequency (VLF) component, LF component, HF component, and total power (TP), respectively.^[16–18]^ It is generally accepted that the efferent vagal activity is a major contributor to HF component fluctuations at the respiratory frequency. Thus, the HF component in the power spectrum of RR intervals is often used to denote the vagal modulation of the patient. By contrast, the LF component in the power spectrum is modulated by both the vagal and sympathetic activities of the patient. Although the interpretation of the ratio of the LF to HF component remains controversial, it is generally used to reflect the balance between sympathetic and vagal modulations. Therefore, the normalized HF component (HF% = 100 × HF/(TP-VLF)) was used as an index of vagal modulation, the LF and LF% components (LF% = 100 × LF/(TP-VLF)) were used as indices of sympathetic and vagal modulation, respectively, and the LF/HF was used as an index of sympathovagal balance.^[16–18]^ The 7 frequency domains (TP, VLF, LF, HF, LF%, HF%, and LF/HF) of the HRV analysis denote different clinical value and autonomic nervous modulation; therefore, the 7 variables have been defined as the priori primary analysis of HRV measures in the present study to explore if they could identify sepsis in patients with suspected infection.

### 2.5 Statistical analyses

Chi-square tests or Fisher exact tests, where appropriate, were used for the statistical analysis of categorical variables. Continuous variables are presented as the mean (standard deviation) and compared using the independent sample *t* test because a normal distribution was noted. The forward selection multiple logistic regression model was used to identify factors that might be associated with sepsis in these patients. Clinical variables and spectral powers of HRV with a univariate comparison of *P* < .2 between the 2 groups were eligible for inclusion in the model. The receiver operating characteristic (ROC) curve for statistically independent HRV variables associated with sepsis was also drawn. A *P* < .05 was considered statistically significant. Bonferroni correction was performed, and *P* < .007 was considered statistically significant for HRV measure comparisons. Statistical analyses were performed using SPSS software system, version 23.0 (IBM, Armonk, NY, USA).

## 3 Results

**Figure 1:**
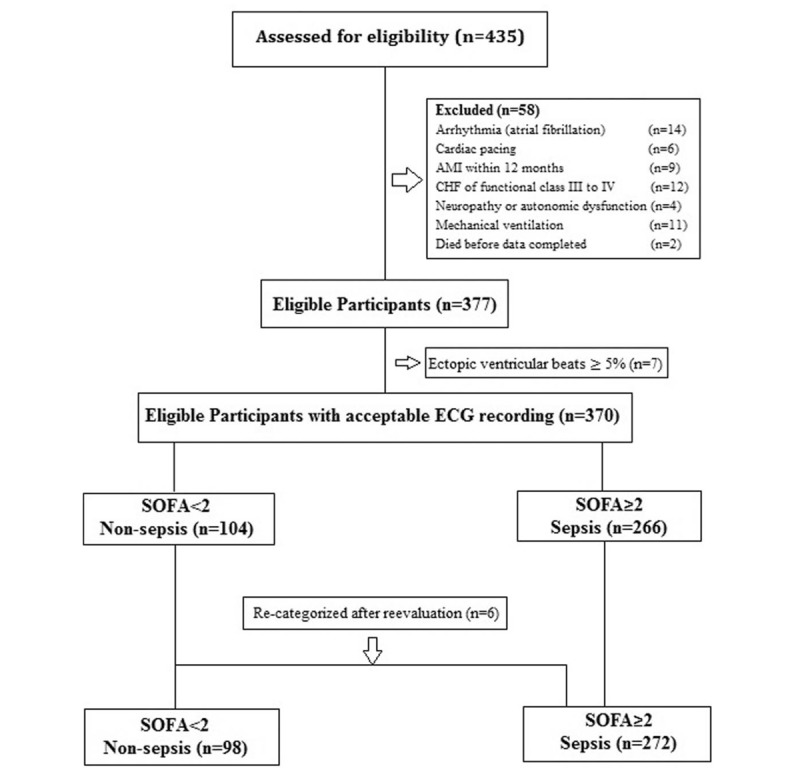
Inclusion flow of the study. AMI = acute myocardial infarction, CHF = congestive heart failure, ECG = electrocardiography, SOFA = Sepsis-related Organ Failure Assessment.

The inclusion flow of the study is depicted in Figure 1. During the 1-year study period, 435 adult patients with suspected infection were treated in the ED. In total, 370 of 435 patients were included in the final analysis. On the basis of the initial and reevaluated SOFA score of ≥2 in the ED, those 370 patients, aged 22 to 84 years, were stratified into non-sepsis (n = 98) and sepsis (n = 272) groups. The mean time from visiting ED to the diagnosis of non-sepsis or sepsis was 92 minutes, with a standard deviation of 16 minutes.

The basic characteristics of both the groups of patients are shown in Table 1. No significant differences were observed in age, sex, ECG recording time, and underlying diseases between these 2 groups of patients. However, the incidence of respiratory tract infection was significantly higher in the non-sepsis group than in the sepsis group.

**Table 1:**
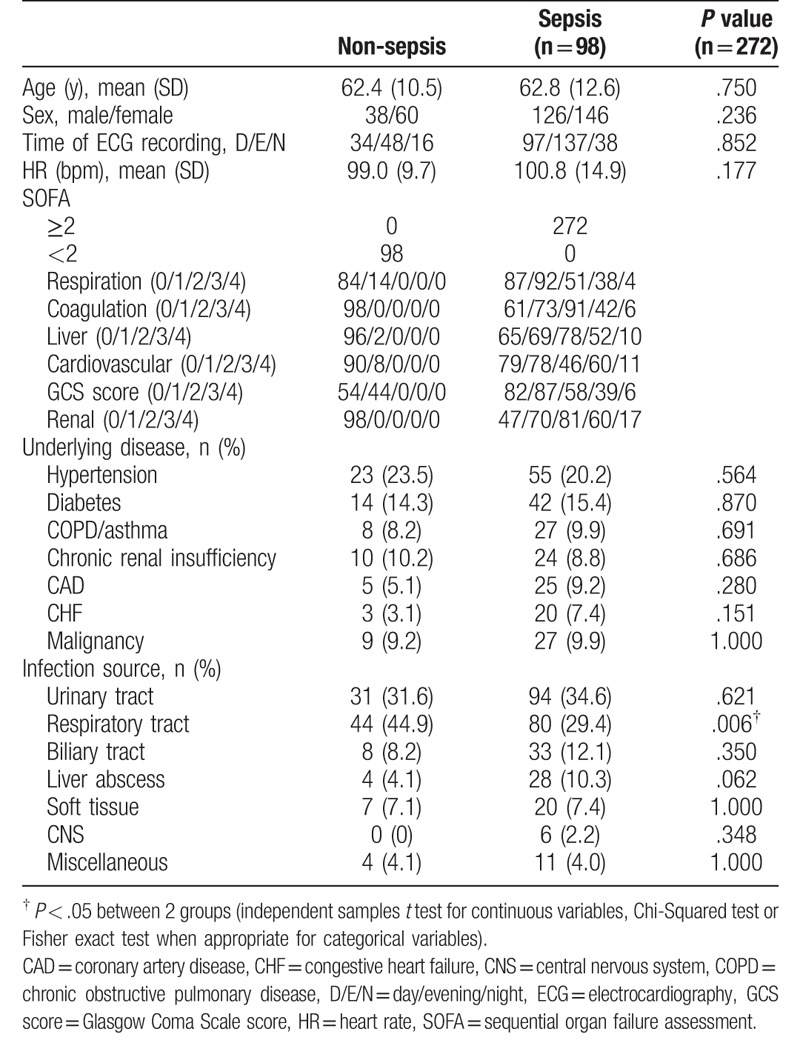
General characteristics of the patients with suspected infection.

Table 2 lists the frequency domains of the HRV measures of both the groups of patients. TP, VLF, HF, and HF% were significantly higher, whereas LF% and LF/HF were significantly lower in the sepsis group than in the non-sepsis group.

**Table 2:**
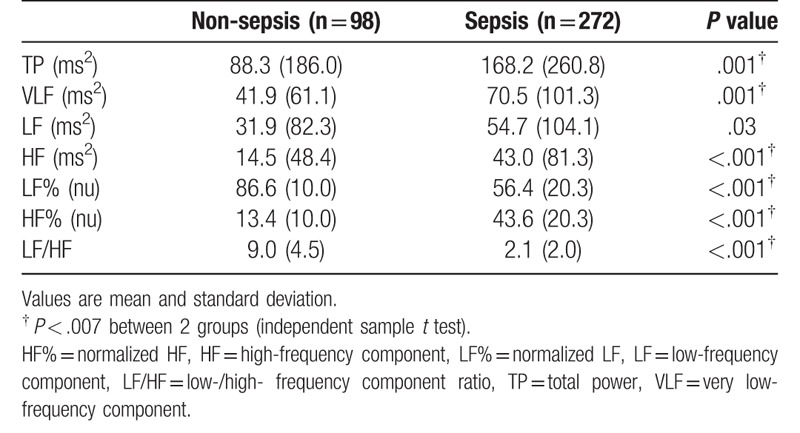
Heart rate variability measures of the patients.

Multiple logistic regression model analysis was performed to analyze independent factors for sepsis. Independent variables included in the analysis were heart rate, CHF, respiratory tract infection, liver abscess, TP, VLF, LF, HF, LF%, HF%, and LF/HF. Results showed that HF component and LF/HF were significantly independent factors for sepsis in adult patients with suspected infection. Odds ratios (95% confidence intervals and *P* values) for HF component and LF/HF were 0.994 (0.990–0.999, .130) and 0.494 (0.423–0.578, < .001), respectively. In addition, LF/HF correlated significantly and negatively with the SOFA score (*r* = −0.505, *P* < .001).

**Figure 2:**
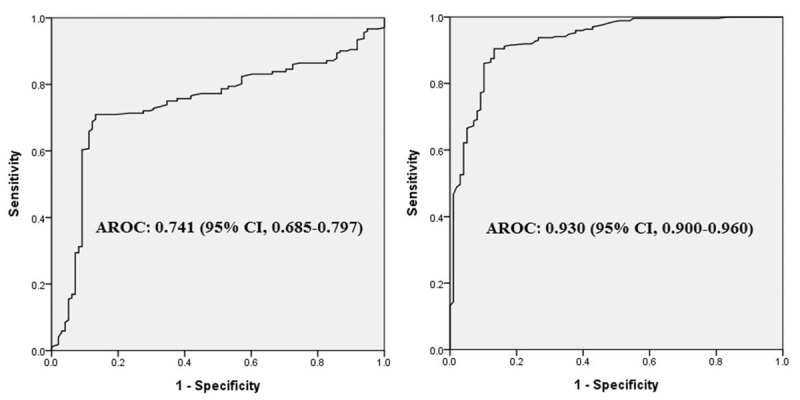
The ROC curves of HF (left) and LF/HF (right) in identifying sepsis in patients with suspected infection. The area under the curves (AROC) for HF and LF/HF were 0.741 (95% CI, 0.685–0.797) and 0.930 (95% CI, 0.900–0.960), respectively.

As depicted in Figure 2, the ROC curves of HF and LF/HF in identifying sepsis were constructed, and the area under the curves were 0.741 (95% CI, 0.685–0.797) and 0.930 (95% CI, 0.900–0.960), respectively. The cut-off value of LF/HF in predicting sepsis was 5.0 with a sensitivity of 90.4% and a specificity of 86.7%.

## 4 Discussion

By performing the power spectral analysis of HRV, in the present study, we found that tilted sympathovagal balance toward increased vagal activity and sympathetic suppression (represented by HF component and LF/HF) could indicate sepsis in patients with suspected infection. In this study, we found that TP, VLF, HF, and HF% were significantly high, whereas LF% and LF/HF were significantly low in patients with sepsis than in patients with non-septic infection. Spectral HRV analysis allows us to differentiate between branches of the autonomic nervous system^[16–18]^ and to assess the autonomic modulation of critically ill patients.^[23]^ HF and HF% are commonly used as indices of cardiac vagal activity.^[16–18]^ Although LF% has been identified with both sympathetic and vagal modulation, they have been noted to have a strong correlation with the sympathetic branch in critically ill patients.^[24,25]^ LF/HF has been reported as the index of sympathovagal balance.^[16,17]^ These results suggest that sepsis patients have increased cardiac vagal activity and tilted sympathovagal balance toward sympathetic suppression compared with the patients without septic infection.

Sepsis is characterized by a complex network of mediators and toxins that might influence cardiovascular reflexes,^[25]^ leading to an impairment of autonomic balance.^[26,27]^ Numerous experimental animal and clinical studies have reported that reduced sympathovagal balance and sympathetic activity, represented by decreased LF/HF and LF%, is a common feature of sepsis.^[20,21,28,29]^ Physiologic host response to counterbalance endotoxin-induced systemic inflammation can result in increased vagal tone^[26]^ and further result in decreased sympathovagal balance. Korach et al reported that a decrease in sympathovagal modulation may precede the sepsis onset for patients on the day after ICU admission.^[29]^ Furthermore, Annane et al indicated that the onset of septic shock was characterized by impaired sympathovagal modulation.^[20]^ The present study indicates that the power spectral analysis of HRV, especially LF/HF, could identify sepsis in patients with suspected infection.

The delivery of critical care is traditionally considered synonymous with ICU care. However, more recently, critically ill patients are cared for in the ED with increasing frequency. The care provided to critically ill patients during their ED stay significantly affects patients’ outcome,^[5]^ suggesting that a practical and available diagnostic tool should be developed in the ED. However, the ICU-based SOFA scoring system, an indispensable part of Sepsis-3,^[6,10]^ is comprehensive and typically requires information that may not be readily available to a physician in the ED. In this study, we found that HF component and LF/HF, the frequency domains of HRV, may be indicators of sepsis in patients with suspected infection as well as the SOFA score, although the exact physiological mechanisms responsible for various HRV components are still incompletely understood. HRV analysis requires only a 5- to 10-minute ECG recording and software that can output HRV measures in 1 to 2 minutes. A 1- to 2-hour training session is generally enough to familiarize physicians with the software and provide them with a readily available tool to identify sepsis in patients with suspected infection.

This study has the following limitations. First, HRV analysis cannot be used to assess patients with non-sinus rhythm,^[16]^ a significant degree of arrhythmia, and cardiac pacing. Second, HRV analysis may not be applicable to patients with CHF with functional class III to IV and recent (within 1 year) AMI. Studies have reported that HRV measures progressively decreased with increasing symptom severity as assessed using the functional class and could identify patients with different severity of heart failure.^[16,30]^ Furthermore, Panina et al demonstrated that significantly higher HRV measures existed in patients with CHF with functional class II than in those with functional class III.^[31]^ Moreover, studies have suggested that HRV decreased in patients with AMI and started to recover several weeks after AMI, but HRV could not fully recover by 12 months.^[16]^ To prevent these interferences, patients with AMI within 12 months or CHF with functional class III to IV were excluded from this study. Third, we excluded patients with neuropathy or autonomic dysfunction that may have significantly impaired autonomic nervous control of the cardiovascular system.^[16]^ Fourth, because the respiratory rate can affect the spectral power,^[16]^ especially the HF, those patients with respiratory failure and on mechanical ventilator support during ECG recording were not included in this study. These restrictions may limit the applicability of the results of the present study to the broader spectrum of patients with sepsis in the ED. In addition, the present results might vary based on the infection type, especially respiratory tract infection combined with tachypnea. We did not attempt to control the respiratory rate of patients because such maneuver would inevitably interfere with the autonomic nervous system of patients, leading to unreal and biased HRV. If patients had slight tachypnea because of respiratory tract infection and even hypoxemia, it would be natural to record and analyze the rhythm of their heart beating as it is. Moreover, patients’ heart rate was not specifically altered or controlled. Tachycardia could lead to confounding alterations in HRV measures. Patients who were currently taking heart rate-altering medication, such as anti-arrhythmia or antihypertensive medication, on their arrival at the ED were not excluded from this study. However, no significant difference was observed in the number of patients with hypertension between the non-sepsis and sepsis groups (Table 1). Apparently, the use of this medication did not significantly affect the results of this study.

## 5 Conclusions

The study demonstrated that patients with sepsis are characterized by tilted sympathovagal balance toward increased vagal activity and depressed sympathetic modulation; however, whether the changes precede sepsis development or vice versa is still uncertain. The HF component and LF/HF of HRV measures may be related to sepsis occurrence in patients with infection and provide clinical physicians additional information on sepsis diagnosis. Given the complexity of sepsis, models incorporating other demographic data, organ function indicators, clinical evaluation parameters, and more complex multivariable and analytic approaches may provide better diagnostic power and improve performance.

## Acknowledgments

This manuscript was edited by Wallace Academic Editing.

## Author contributions

**Conceptualization:** Wei-Lung Chen

**Data curation:** Ching-Tang Hsu and Wei-Lung Chen

**Formal analysis:** Jiann-Hwa Chen

**Funding acquisition:** Wei-Lung Chen

**Investigation:** Jui-Yuan Chung

**Methodology:** Jiann-Hwa Chen and Wei-Lung Chen

**Resources:** Ching-Tang Hsu, Henry Chih-Hung Tai, Jui-Yuan Chung, Jiann-Hwa Chen, and Wei-Lung Chen

**Software:** Jui-Yuan Chung and Wei-Lung Chen

**Supervision:** Wei-Lung Chen

**Validation:** Jiann-Hwa Chen and Wei-Lung Chen

**Visualization:** Jui-Yuan Chung

**Writing – original draft:** Ching-Tang Hsu

**Writing – review & editing:** Henry Chih-Hung Tai and Wei-Lung Chen

Wei-Lung Chen orcid: 0000-0002-6074-3872.
